# The Potential of ChatGPT for High-Quality Information in Patient Education for Sports Surgery

**DOI:** 10.7759/cureus.58874

**Published:** 2024-04-23

**Authors:** Ali Yüce, Nazım Erkurt, Mustafa Yerli, Abdulhamit Misir

**Affiliations:** 1 Department of Orthopedics and Traumatology, Prof. Dr. Cemil Taşçıoğlu City Hospital, Istanbul, TUR; 2 Department of Orthopedics and Traumatology, Bahcesehir University Göztepe Medicalpark Hospital, Istanbul, TUR

**Keywords:** orthopedic sports medicine, patient education, sports surgery, chatbots, chatgpt, artificial intelligence(ai)

## Abstract

Background and objective: Artificial intelligence (AI) advancements continue to have a profound impact on modern society, driving significant innovation and development across various fields. We sought to appraise the reliability of the information offered by Chat Generative Pre-Trained Transformer (ChatGPT) regarding diseases commonly associated with sports surgery. We hypothesized that ChatGPT could offer high-quality information on sports-related diseases and be used in patient education.

Methods: On September 11, 2023, specific sports surgery-related diseases were identified to ask ChatGPT-4 (personal communication, March 4, 2023). The informative texts provided by ChatGPT were recorded by a non-observer senior orthopedic surgeon for this study. Ten texts provided by ChatGPT related to sports surgery diseases were evaluated blindly by two observers. Observers assessed and scored these texts based on the sports surgery-specific scoring (SSSS) and DISCERN criteria. The precision of the disease-related information offered by ChatGPT was evaluated.

Results: The calculated average DISCERN score of the texts in the study was 44.75 points and the average SSSS score was 13.3 points. In the interclass correlation coefficient analysis of the measurements made by the observers, the agreement was found to be excellent (0.989; p < 0.001).

Conclusion: ChatGPT has the potential to be used in patient education for sports surgery-related diseases. The potential to provide quality information in this regard seems to be an advantage.

## Introduction

Artificial intelligence (AI) advancements have resulted in significant innovations and developments across various fields [1]. Chatbots are software applications designed to replace human interaction by facilitating online conversational communication [2]. Chat Generative Pre-trained Transformer (ChatGPT; OpenAI, United States) is an AI platform introduced to the public in November 2022. This technology, capable of generating human-like text, is being considered as a tool that could reduce the workload of writing scientific journal articles while maintaining academic writing standards [3].

Cutting-edge computer technologies and techniques, such as chatbots and AI applications, can be used to provide patients and their families with the information they need about diseases and treatments [2,4,5]. With the recent acceleration in the development of AI language models like ChatGPT, we can expect significant changes in how medical students and patients access and interact with information [6]. In general, ChatGPT has the potential to be a tool for patient education and participation. ChatGPT can provide patients with critical information about their health and potential outcomes, thus reducing anxiety and potentially improving outcomes [3]. However, ensuring the standardization, reliability, and integrity of the information generated by ChatGPT in specific domains is of vital importance [6].

The aim of this study was to evaluate the quality of information offered by ChatGPT regarding diseases associated with sports surgery. We hypothesized that ChatGPT could offer high-quality information on sports-related diseases and be used in patient education.

## Materials and methods

Specific sports surgery-related diseases were identified on September 11, 2023. Open AI's ChatGPT-4 (personal communication, March 4, 2023) was utilized in this investigation. First, we initiated a chat on ChatGPT with the input “Can you provide a high-quality informative text about XXX and its surgery?” Texts were created by writing diseases related to sports surgery in the place specified as XXX (for example, Bankart lesion). The identified diseases were as follows: 1) rotator cuff rupture, 2) Bankart lesion, 3) frozen shoulder, 4) anterior cruciate ligament rupture, 5) meniscus tear, 6) posterior cruciate ligament rupture, 7) femoroacetabular impingement, 8) talus osteochondral lesion, 9) ankle ligament tears, and 10) lateral epicondylitis. Subsequently, a new conversation was started with each text request. The educational text associated with each of the diseases related to sports surgery was requested from ChatGPT in a new chat. Then, 10 educational texts prepared by ChatGPT were recorded.

The informative texts provided by ChatGPT were recorded by a non-observer senior orthopedic surgeon for this study.

The website for patient education of the American Academy of Orthopaedic Surgeons, OrthoInfo.org, is one of several reputable orthopedic sources available on the web [7]. Therefore, disease information texts related to the identified sports surgery-related diseases on the OrthoInfo website were evaluated. Subsequently, SSSS criteria were established based on these texts found on OrthoInfo. The SSSS criteria are summarized in Table 2. The SSSS consisted of four subheadings: disease presentation, diagnostic process, treatment, and postoperative period. The SSSS comprised 20 criteria, with a maximum score of 20. The text was assessed according to the specified criteria, utilizing a scoring system graded as very poor (0-4 point), poor (5-8 point), fair (9-12 point), good (13-16 point), and excellent (17-20 point). This approach has been employed in prior research [8,9].

The DISCERN has 16 questions in three sections, with higher scores indicating better quality [10] (Table 3). The first eight questions assess reliability, while the following seven evaluate specific treatment details. The final question pertains to the overall quality of the publication. Total points indicated excellent (63-75 points), good (51-62 points), fair (39-50 points), poor (27-38 points), and very poor (16-26 points) quality.

Ten texts provided by ChatGPT related to sports surgery diseases were evaluated blindly by two observers. Observers assessed and scored these texts based on the SSSS and DISCERN criteria. Subsequently, the scores obtained for the texts provided by ChatGPT were evaluated, and interobserver agreement was assessed. The quality of the information provided by ChatGPT about these diseases was evaluated.

Inter-observer agreements were investigated using interclass correlation coefficient statistics for continuous data.

Statistical analysis

The results can be interpreted as follows: agreement was poor for values below 0.50, moderate for values ranging from 0.50 to 0.75, good for values ranging from 0.75 to 0.90, and excellent for values ranging from 0.90 to 1.00. The statistical analysis was conducted using IBM SPSS Statistics for Windows, Version 25 (Released 2017; IBM Corp., Armonk, New York, United States), and the level of statistical significance was set at p < 0.05.

## Results

The mean DISCERN score of the texts included in the study was calculated as 45 (range: 39-52), and the mean SSSS score was 13.6 (range: 10-17). In the interclass correlation coefficient analysis of the measurements made by the observers according to the DISCERN criteria, the agreement was found to be good (ICC: 0.717; p: 0.046). In the interclass correlation coefficient analysis of the measurements made by the observers according to the SSSS criteria, the agreement was found to be excellent (ICC: 0.922; p: 0.001). The values of the texts according to the diseases are summarized in Table 1. In the analysis made by taking the average of the evaluations of the two observers in the study, when the categories of the scores received according to the DISCERN criteria were examined, it was seen that all of them were in the fair quality category. Likewise, according to the SSSS criteria, 60% of the scores received were categorized as good quality and the remaining were categorized as fair quality.

**Table 1:** Scores of the texts examined in the study from observers * indicates statistical significance. SSSS: sports surgery-specific scoring, ICC: intraclass correlation coefficient, ACL: anterior cruciate ligament, PCL: posterior cruciate ligament

	DISCERN				SSSS			
	Observer-I		Observer-II		Observer-I		Observer-II	
	Point	Category	Point	Category	Point	Category	Point	Category
Rotator cuff rupture	49	Fair	49	Fair	15	Good	15	Good
Bankart lesion	39	Fair	40	Fair	10	Fair	12	Fair
Frozen shoulder	50	Fair	50	Fair	16	Good	16	Good
ACL rupture	39	Fair	41	Fair	15	Good	15	Good
Meniscus tear	47	Fair	40	Fair	12	Fair	10	Fair
PCL rupture	46	Fair	48	Fair	15	Good	15	Good
Femoroacetabular impingement	42	Fair	48	Fair	14	Good	12	Fair
Talus osteochondral lesion	43	Fair	44	Fair	12	Fair	12	Fair
Ankle ligament tear	44	Fair	44	Fair	11	Fair	12	Fair
Lateral epicondylitis	52	Good	45	Fair	16	Good	17	Good
ICC	0.717				0.922			
p	0.046*				0.001*			

## Discussion

One of the key findings of this study was that ChatGPT provided moderately to good quality informational texts on sports surgery according to both DISCERN (100% fair) and SSSS (60% good, 40% fair). The way people access information has changed significantly with laptops, tablets, and smartphones [11]. Orthopedic patients often rely on the Internet to gather information about their conditions [12,13]. Patients attempting to obtain information about their illnesses via the Internet raised concerns about accessing incorrect information [14]. Therefore, the quality of sports surgery-related diseases on the internet and social media platforms was investigated, and the majority of them contained low-quality information [15-20]. Chatbots like ChatGPT are likely to become one of the new sources of information for patients [2-4,21]. Since its launch, ChatGPT has established its presence in higher education. This chatbot supports over 40 languages and excels in creating human-like dialogues [22]. Being available online and open to everyone, ChatGPT has the potential to become a new reference source for orthopedic patients, similar to the Internet and social media [3]. Therefore, it is crucial to assess the accuracy of the information generated by ChatGPT in the same way that the quality of information on the internet and social media platforms is assessed. According to the results of this study, ChatGPT has the potential to be a good source of information for patients.

The Internet is largely unregulated, and there is potential for patients to find incorrect or misleading information about their health conditions or treatment options [23]. ChatGPT, on the other hand, can provide patients with critical information about their health and potential outcomes, reducing anxiety and potentially improving outcomes. However, considering the possible oversight of important references and current research, ChatGPT in this format is thought to potentially pose a danger [3]. Nevertheless, ChatGPT provided many essential subheadings in this study. On the other hand, it provided brief and not highly detailed information. In this study, the main text provided by ChatGPT was evaluated, and additional questions were not asked. Nevertheless, it provided significant information. It also informed patients about complications or treatment options. Given this, ChatGPT appears to be a tool with high potential for providing quality information to patients. However, a separate study could focus on the questions frequently asked by patients after receiving information and the accuracy and quality of ChatGPT's responses to these questions.

The DISCERN scoring system was jointly developed by employees of the University of Oxford and the British Library and designed for use by healthcare consumers [24]. Approved assessment tools like the DISCERN questionnaire can be used to evaluate written healthcare information [10]. ChatGPT did not receive very high scores in this study according to the DISCERN scoring. In DISCERN, all texts received the lowest scores for questions such as "Is it clear what sources of information were used to compile the publication (other than the author or producer)?" "Is it clear when the information used or reported in the publication was produced?" and "Does it provide details of additional support and information sources?" Since ChatGPT is a chatbot, it seems normal not to provide this information. Furthermore, it cannot be expected to provide additional information sources since it can ask for this or you need to request it. To overcome this issue, modifying DISCERN or developing a scoring system specifically for chatbots in future studies may be necessary.

One limitation of this study is that ChatGPT provides instant responses to the questions asked. In future studies, it must demonstrate that it responds consistently and at the same standards of quality when questions are asked at different times and using different accounts. Additionally, as mentioned earlier, the quality of ChatGPT's responses to frequently asked follow-up questions after the initial information provided to patients has not been evaluated. Orthopedic sports surgery is a broad subject and only the most common diseases are evaluated here. One of the limitations is not including more diseases and not controlling how AI provides information about these diseases. Finally, the SSSS is a scoring system that was used for the first time in this study. Indeed, similar standardized scoring systems have been used in previous studies on the evaluation of patient information [8,9]. Additionally, ChatGPT is an AI service capable of generating text that closely resembles what humans write, but like other statistical models, it is not error-free. Its fundamental limitations are the lack of human-like understanding and the lack of data entry after 2021, which can sometimes result in the generation of irrelevant texts or ideas and concepts that are truly unoriginal or not unique, disregarding the context of the information request [25]. Therefore, although not identified in this study's texts, the possibility of providing erroneous information to patients should not be overlooked.

## Conclusions

ChatGPT produced informational texts of moderate to good quality across various sports surgery topics according to the DISCERN and SSSS criteria. This suggests that ChatGPT could be utilized in patient education for diseases related to sports surgery. The ability to offer quality information consistently appears to be a potential benefit. However, further research is necessary to verify its consistent performance and to predict the accuracy and quality of responses to additional questions from patients.

## Appendices

**Table 2:** Sport surgery-specific scoring Max: Maximum

Diagnostic information	Point
Definition of the disease	max 1 point
Patient population	max 1 point
Complaints and symptoms	max 1 point
Activity limitations	max 1 point
Risk factors	max 1 point
Pathoanatomy of the disease	max 1 point
Diagnostic process	Point
Physical examination	max 1 point
Radiological imaging	max 1 point
Differential diagnosis	max 1 point
Diagnostic process	max 1 point
Treatment	
Conservative treatment	Point
Medical treatment	max 1 point
Brace treatment, injections, and physical therapy	max 1 point
Activity modifications	max 1 point
Surgical treatment	Point
It mentions that surgical treatment may be required in cases where non-surgical treatment fails	max 1 point
Open surgery	max 1 point
Arthroscopic surgery	max 1 point
Complications	max 1 point
Postoperative	Point
Postoperative rehabilitation	max 1 point
Treatment alternatives and processes after unsuccessful surgical treatment	max 1 point
Recovery time refers to the process of returning to normal activities and work	max 1 point

**Table 3:** DISCERN scoring system

Section 1: Is the publication reliable?
1) Are the aims clear?
2) Does it achieve its aims?
3) Is it relevant?
4) Is it clear what sources of information were used to compile the publication (other than the author or producer)?
5) Is it clear when the information used or reported in the publication was produced?
6) Is it balanced and unbiased?
7) Does it provide details of additional sources of support and information?
8) Does it refer to areas of uncertainty?
Section 2: How good is the quality of information on treatment choices?
9) Does it describe how each treatment works?
10) Does it describe the benefits of each treatment?
11) Does it describe the risks of each treatment?
12) Does it describe what would happen if no treatment is used?
13) Does it describe how the treatment choices affect the overall quality of life?
14) Is it clear that there may be more than one possible treatment choice?
15) Does it provide support for shared decision-making?
Section 3: Overall rating of the publication
16) Based on the answers to all of the above questions, rate the overall quality of the publication as a source of information about treatment choices
